# Anesthetic management of a patient with trisomy 18 undergoing esophageal banding and preceding gastrostomy—A case report

**DOI:** 10.1002/ccr3.6404

**Published:** 2022-10-03

**Authors:** Misaki Kano, Daisuke Sugiyama, Kenichi Ueda, Osamu Kobayashi

**Affiliations:** ^1^ Department of Anesthesiology Kameda Medical Center Kamogawa City Japan

**Keywords:** 18 trisomy, complex congenital heart disease, double‐outlet right ventricle, esophageal banding, tracheaesophageal fistula

## Abstract

A patient diagnosed with trisomy 18 is at great risk of perioperative morbidity and mortality, especially with complex congenital cardiac anomalies. Here, we report successful anesthetic management of palliative esophageal‐banding and gastrostomy for trachea‐esophageal fistula in a patient who had a complex congenital heart disease with trisomy 18.

## Abstract 2

Ventilatory management of patients with type C tracheoesophageal fistula and its impact on the hemodynamic/saturation status of double‐outlet right ventricle for trisomy 18 patients.

## 1 INTRODUCTION

Trisomy 18 was first described by Edwards et al.^1^ in the 1960s. Its incidence is one in 8000 live births, but the prognosis is extremely poor, with a 90% death rate within the first 12 months of age. It is characterized by multiple malformations such as structural heart defects, microcephaly, micrognathia, low‐set ears, overlapping fingers, gastrointestinal, and genitourinary system defects. Traditionally, surgical procedures are not indicated for patients with trisomy 18 due to a high mortality rate in the perinatal period. However, more recently, intensive care of those patients shows improved outcomes. We report the anesthetic management in a patient with trisomy 18 undergoing esophageal banding and gastrostomy for esophageal atresia with tracheoesophageal fistula (TEF) for the preparation of cardiac surgery. The patient's family has provided “written consent” to publish this case report.

## 2 CASE

**FIGURE 1:**
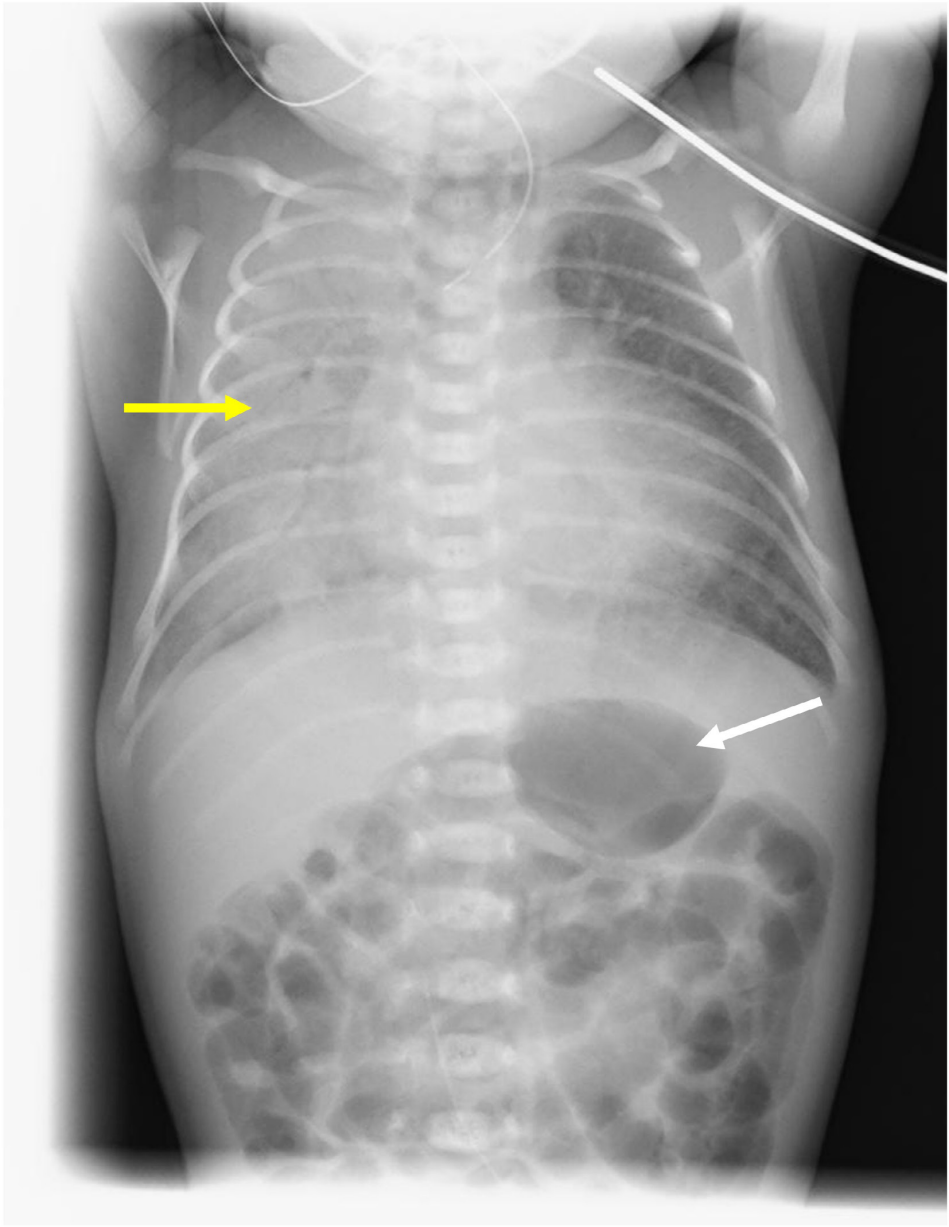
A preoperative chest X‐ray. The stomach was dilated with gas (white arrow), and the right lung had decreased X‐ray permeability because of the atelectasis (yellow arrow).

A 4‐day‐old female infant was scheduled to undergo esophageal banding and gastrostomy for type‐C esophageal atresia. Trisomy 18 had been diagnosed prenatally, and she was born at the 38th gestational week with a birth weight of 1498 g. This infant had a double‐outlet right ventricle (DORV) with patent foramen ovale (PFO) and a large ventricular septal defect (VSD), leading to moderate pulmonary hypertension. Echocardiography showed normal wall motion, VSD (left to right shunt) and PFO (left to right shunt), 2.2 mm of patent ductus arteriosus (PDA). Results of tests for blood cell counts, urea, creatinine, electrolytes, and liver function were within normal limits. A chest x‐ray demonstrated decreasing x‐ray‐permeability of the right lung because of the atelectasis (Figure 1). The atelectasis was thought to be due to lung immaturity and ventilatory management immediately after birth. She was managed in the neonatal intensive care unit (NICU) after delivery and was maintained on nasal continuous positive airway pressure (CPAP: The flow is 9 L/min for expiration and + 3 L/min for inspiration.) in room air with pulse oxygen saturation (SpO_2_) 88%–99%. No gastric distention due to nasal CPAP was noted. Her pulse rate was maintained around 130–170 beat per minute (bpm). Other than low‐set ears, there was no facial abnormality, which could have caused a difficult airway. The parents were not considering surgery or any advanced treatment before the baby was born because the long‐term prognosis was not promising. However, they strongly wished to go home with the child after birth, even if for a short period of time. For this reason, TEF closure and a feeding gastrostomy were performed on the fourth day after birth for palliative purposes and to fulfill the parents' wishes.

On the day of surgery, her weight was 1426 g. Pulse oxygen saturation was above 90% on FiO_2_: 0.21 with nasal CPAP. Electrocardiography showed a sinus rhythm of 128 bpm. After application of ASA standard monitors, radial arterial blood pressure, and cerebral and somatic oximetry, the patient was preoxygenated with 100% oxygen. General anesthesia was induced with 0.1 mg/kg midazolam and 2 μg/kg fentanyl. Muscle relaxation was facilitated by 1.0 mg/kg rocuronium bromide with the surgical team on standby for immediate gastrocentesis. In order to minimize gastric insufflation, pressure was applied on the upper abdomen during mask ventilation. The trachea was intubated with an endotracheal tube (size 3.0 mm uncuffed oral tube) without difficulty. After tracheal intubation, the intubation tube was fixed at the right mouth angle of 8.0 cm after auscultation of bilateral breath sounds. A slight leak was observed at a pressure of 15 cmH_2_O, and there was no prominent exacerbation of gastric distention on ventilation after tracheal intubation. FiO_2_ was returned to 21% promptly after tracheal intubation. Anesthesia was maintained with intermittent administration of 5 μg/kg fentanyl and midazolam 0.1 mg/kg as needed. Fiberoptic bronchoscopy was performed to identify TEF, which was found on the same level as the tracheal carina. The patient was positioned in left lateral decubitus position for right thoracotomy. During surgery, bilateral lung ventilation was maintained with the intention of preventing exacerbation of pulmonary hypertension. FiO_2_ was adjusted to keep SpO_2_ in above 90%. Systemic blood pressure, heart rate, and cerebral and somatic oximetry were stable without any inotropic support throughout the case (Table 1). After esophageal banding, we confirmed that the stomach was not insufflated with gas even though the gastrostomy tube was clamped. After surgery, the patient was kept intubated and was transferred to NICU. Her trachea was extubated in the NICU on the day of surgery. Five weeks later she underwent pulmonary artery banding without sequela. The patient was discharged from the hospital and was able to spend 6 months at home with her family before she died.

**TABLE 1:** Blood pressure (BP), heart rate (HR), SpO_2_ and FiO_2_ during surgery

	When entering the room	Induction of anesthesia	Incision	After esophageal banding	When the surgery was over
sBP/dBP (mmHg)	69/43	73/45	70/44	68/41	65/42
HR (bpm)	129	150	145	155	155
SpO_2_ (%)	95	91	96	94	95
FiO_2_(%)	21	100	21	21	21

## 3 DISCUSSION

Trisomy 18, also called Edward's syndrome, is the second most common autosomal trisomy after trisomy 21. Approximately 50% of babies with trisomy 18 live longer than 1 week, and only 5%–10% of children survive beyond the first year. Structural heart defects occur in over 90% of infants with this syndrome. The most common cardiac anomalies are ventricular and atrial septal defects, PDA, and valvular disease. More complex malformations, including DORV and endocardial cushion defect, are present in about 10% of cases, which contributes to early mortality. Esophageal atresia with TEF is a significant medical condition, leading to recurrent aspiration pneumonitis.^2^

Anesthetic management for TEF is challenging due to potential ventilatory deterioration. Maintenance of spontaneous ventilation has been recommended for anesthetic management, especially in cases in which a large fistula, interferes with ventilation by gastric overdistention when positive ventilation is applied.^3^, ^4^ Notably, Deanovic et al.^5^ reported that all 47 patients in a case series tolerated positive ventilation without respiratory deterioration. Thus, depending on pulmonary compliance and the size of a fistula, patients with TEF can be managed with positive ventilation. In our case, after discussion with the surgical team, we prioritized controlling ventilation over the risk of gastric insufflation, because controlling ventilation is essential for maintaining a balance between pulmonary and systemic flow in a patients with DORV, and gastric insufflation can be managed with gastrocentesis.

A patient with parallel circulation, such as DORV, requires controlled ventilation in order to balance systemic vascular resistance and pulmonary vascular resistance (PVR). Hypercarbia, hypoxia, and sympathetic stimulus can increase PVR, which causes pulmonary under‐circulation and exacerbates hypoxia. Hypocarbia, and hyperoxia, can decrease PVR, which causes pulmonary overcirculation and systemic hypotension. Thus, it is crucial to monitor systemic oxygen delivery by using cerebral and somatic oximetry, venous oxygen saturation, and lactate level for judicious titration in the ventilation setting. In our case, close communication with the surgical team allowed us to keep SpO_2_ at above 90% with minimum increase in oxygen content. Systemic blood pressure and cerebral and somatic oximetry were stable and required no intervention.

Historically, there has been a consensus among care providers that trisomy 18 be considered a condition for which no intervention in the newborn is indicated, because of the elevated risk of mortality in the first month of life and the presence of significant developmental disability in surviving children.^6^ However, some recent reports have indicated that intensive care for those trisomy 18 infants, including cardiovascular operation, could lead to a better prognosis.^2^ Thus, it is likely that number of these patients requiring anesthetic management will increase.

Anesthetic management for trisomy 18 is uncommon and challenging. Multiple comorbidities, including cardiovascular malformations, complicate anesthesia management in this population. In this report, we described our experience with the management of an 18 trisomy patient with TEF. Individualized anesthetic management according to the patient's condition and close communication with a surgical team are keys for successful intraoperative management.

## AUTHOR CONTRIBUTIONS

Misaki Kano: This author had the case and helped data collection and prepared the first manuscript. Daisuke Sugiyama: This author helped data collection, prepared the first manuscript, and edited the manuscript. Kenichi Ueda: This author reviewed and edited the manuscript. Osamu Kobayashi: This author had the case and edited the manuscript.

## CONFLICT OF INTEREST

None.

## CONSENT

Patient parents agree to publish this case report and written consent has been got.

## Data Availability

Data available on request due to privacy/ethical restrictions.
